# SynechoNET: integrated protein-protein interaction database of a model cyanobacterium *Synechocystis *sp. PCC 6803

**DOI:** 10.1186/1471-2105-9-S1-S20

**Published:** 2008-02-13

**Authors:** Woo-Yeon Kim, Sungsoo Kang, Byoung-Chul Kim, Jeehyun Oh, Seongwoong Cho, Jong Bhak, Jong-Soon Choi

**Affiliations:** 1 Korean BioInformation Center, KRIBB, Daejeon 305-806, Korea; 2 Proteomics Team, Korea Basic Science Institute, Daejeon 305-333, Korea

## Abstract

**Background:**

Cyanobacteria are model organisms for studying photosynthesis, carbon and nitrogen assimilation, evolution of plant plastids, and adaptability to environmental stresses. Despite many studies on cyanobacteria, there is no web-based database of their regulatory and signaling protein-protein interaction networks to date.

**Description:**

We report a database and website SynechoNET that provides predicted protein-protein interactions. SynechoNET shows cyanobacterial domain-domain interactions as well as their protein-level interactions using the model cyanobacterium, *Synechocystis *sp. PCC 6803. It predicts the protein-protein interactions using public interaction databases that contain mutually complementary and redundant data. Furthermore, SynechoNET provides information on transmembrane topology, signal peptide, and domain structure in order to support the analysis of regulatory membrane proteins. Such biological information can be queried and visualized in user-friendly web interfaces that include the interactive network viewer and search pages by keyword and functional category.

**Conclusion:**

SynechoNET is an integrated protein-protein interaction database designed to analyze regulatory membrane proteins in cyanobacteria. It provides a platform for biologists to extend the genomic data of cyanobacteria by predicting interaction partners, membrane association, and membrane topology of *Synechocystis *proteins. SynechoNET is freely available at  or directly at .

## Background

Cyanobacteria are prokaryotic microorganisms that perform plant-like photosynthesis as well as carbon and nitrogen assimilation to obtain intracellular energy. Since cyanobacteria are believed to be a prototype organism that changed the ancient anoxygenic environment to oxygenic by photosynthesis, many scientists have used cyanobacteria as an ideal model organism to study adaptation to various abiotic environmental stress [1]. Furthermore, cyanobacteria are capable of producing renewable energy source and sequestering carbon dioxide which causes global warming [2]. The entire genome sequence of the unicellular cyanobacterium *Synechocystis *sp. PCC 6803 (henceforth referred to as *Synechocystis*) was determined at Kazusa DNA Research Institute [3]. The sequence and annotation information is served at an online genome database named CyanoBase [4], which also provides CyanoMutants, a repository with cyanobacterial mutant information. As well as in genomics, transcriptomics, proteomics, and metabolomics fields [3,5-8], *Synechocystis *has been highlighted to integrate "omics" data in systems biology field [9]. However, little has been attempted in the field of interactomics. In particular, there is no web-based database of their regulatory and signaling protein-protein interaction networks to date.

**Figure 1:**
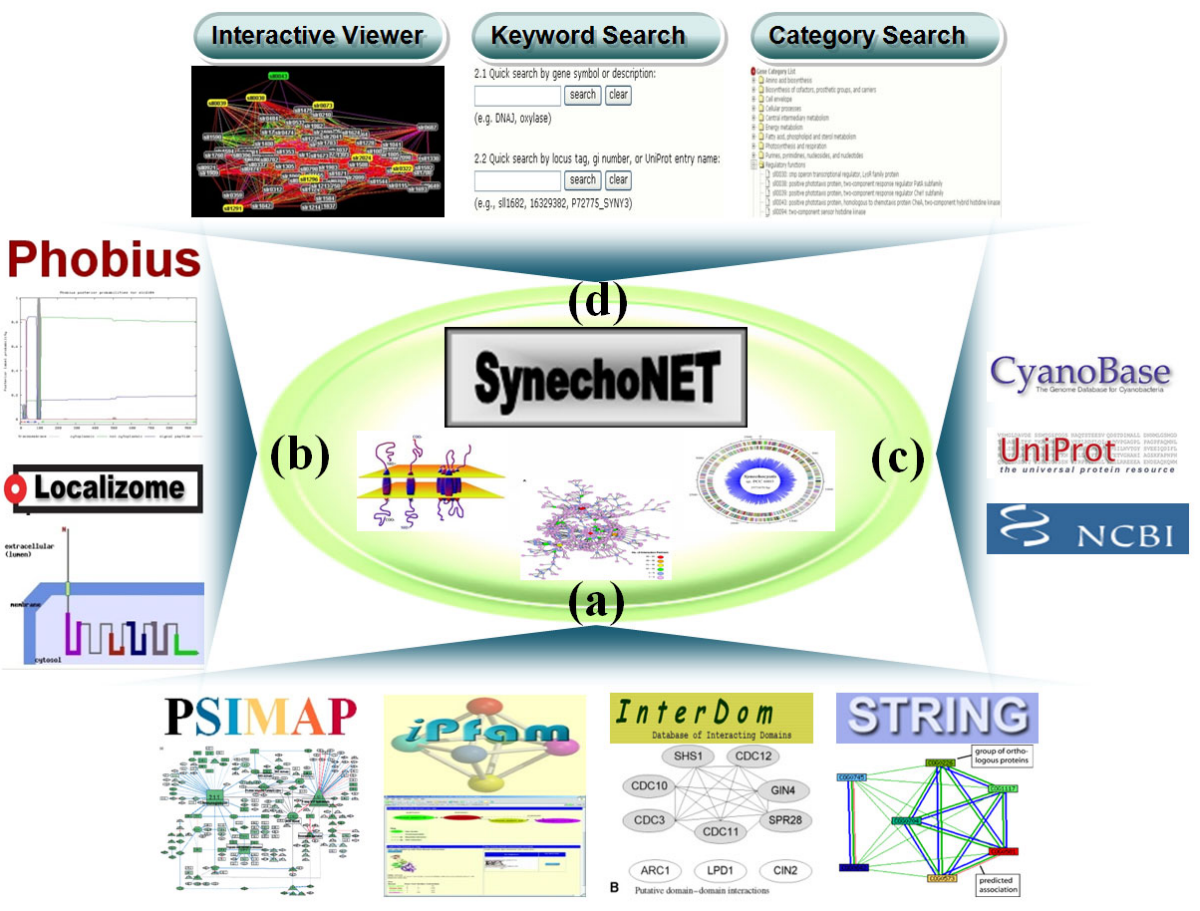
**Overview of the SynechoNET system**. SynechoNET provides four kinds of information or components: (a) integrated protein-protein interaction information from PSIMAP, iPFAM, InterDom, and STRING, (b) protein information regarding transmembrane topology and domain structure based on Phobius and Localizome programs, (c) external links to public databases such as CyanoBase, UniProt, and NCBI, and (d) user-friendly web interfaces such as an interactive network viewer and search pages by keyword and functional category.

**Figure 2:**
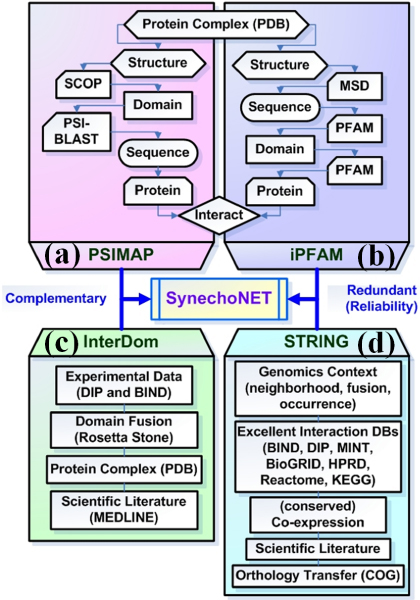
**Integration of public protein-protein interaction data**. SynechoNET integrates four protein-protein interaction databases that contain mutually complementary as well as redundant information. (a) PSIMAP provides protein-protein interaction data based on PDB, SCOP, and PSI-BLAST search. (b) Likewise, iPFAM provides protein-protein interaction data based on PDB, MSD, and PFAM. (c) On the other hand, InterDom is a collective database of putative interacting protein domains from experimental data, domain fusion, protein complex, and scientific literature. (d) Similarly, STRING integrates protein-protein interaction data from various data sources such as genomics context, curated interaction databases, conserved co-expression, and scientific literature. Furthermore, it expands those protein-protein interactions through orthology transfer originally based on the COG database.

**Figure 3:**
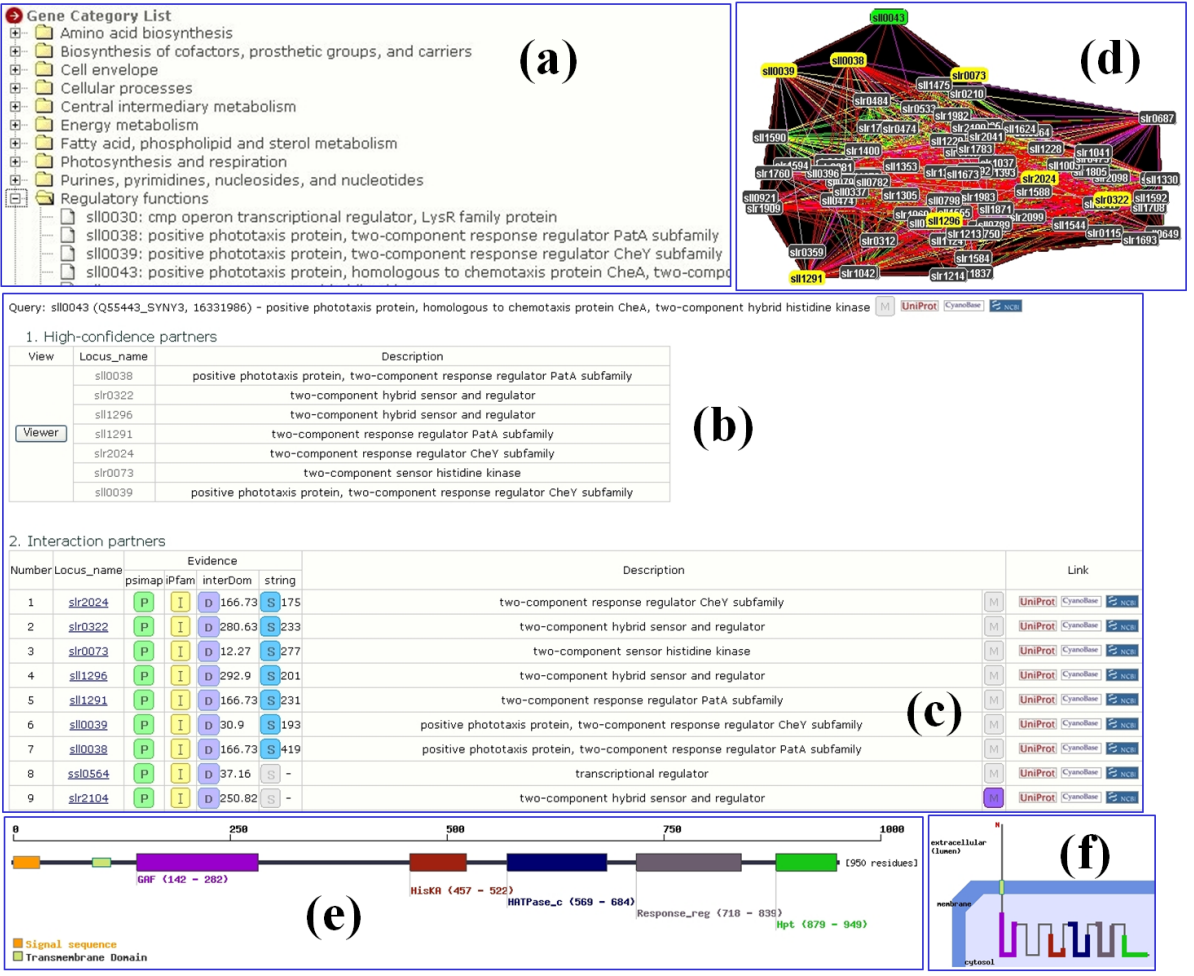
**Web interfaces of SynechoNET**. SynechoNET provides various user-friendly web interfaces and useful biological information. For instance, when users click 'sll0043' from category search page (a), the users can get a search result page displaying a list of highly reliable interaction partners of the clicked protein (b) as well as the candidate proteins predicted to interact with sll0043-encoded protein (c). When the users click the 'viewer' button in the search result page, a web page containing interactive Java applet viewer pops up to display the interaction network containing the selected protein (green box) and its interacting partners (yellow box) (d). Moreover, when the users click the 'M' button (violet box) in the search result page indicating a membrane protein, the information on protein domain structure (e) and transmembrane topology (f) of the selected protein, 'slr2104' will be displayed by Phobius and Localizome programs.

Thus, we developed a database, SynechoNET, which is a protein-protein interaction database for *Synechocystis *(Figure 1). We integrated four public protein-protein interaction databases, namely, Protein Structural Interactome map (PSIMAP) [10], iPfam [11], InterDom [12], and STRING [13] (Figure 1a and 2). The four protein-protein interaction databases provide their own structure-based or integrated interaction data by using different strategies and resources. PSIMAP is a global and general interaction map that provides structural interaction and interacting interface information for known Protein Data Bank (PDB) [14] structures. It calculates the Euclidean distance to determine possible pairs of structural domains in proteins, based on the 5-5 rule [10]. To predict protein-protein interactions, PSIMAP first derives domain-domain interactions from structural interaction data of PDB via SCOP [15]. The domain-domain interactions are then expanded to protein-protein interactions via the sequence similarity search using PSI-BLAST [16] (Figure 2a). Likewise, iPfam is a resource that describes domain-domain interactions among Pfam [17] domains observed in PDB entries. It derives the domain-domain interactions from PDB via the MSD database [18] which provides UniProt [19] to PDB mapping [11]. The derived domain-domain interaction is finally expanded to protein-protein interaction through Pfam. On the other hand, InterDom [12] is a collective database of putative interacting protein domains from domain fusion [20], Database of Interacting Proteins (DIP) [21], Biomolecular Interaction Network Database (BIND) [22], PDB, and MEDLINE [23] (Figure 2c). Similarly, STRING is an integrated database with known and predicted protein-protein interaction information derived from genomics context, high-throughput experiments, conserved co-expression, previous knowledge data, and curated interaction databases [13] (Figure 2d). Furthermore, those interaction data are further expanded through orthology transfer originally based on the COG database [24]. Since the above four databases are complementary as well as redundant to generate the possible protein-protein interactions, SynechoNET is allowed to specify the interacting protein pairs of *Synechocystis *at various levels of confidence and sensitivity. In particular, *in silico *redundant interacting domain information derived from PSIMAP, iPfam, and InterDom gives useful clues for predicting and assigning the function of unknown or hypothetical proteins. Furthermore, SynechoNET provides information on transmembrane topology, signal peptide, and domain structure available from Phobius [25] and Localizome [26], which enable us to analyze regulatory membrane proteins (Figure 1b, 3e, and 3f). This module can be useful to interpret the unknown regulation of membrane proteins linked to significant functions of cyanobacteria, such as photosynthesis, respiration, and two-component signal transduction. In addition, SynechoNET provides helpful hypertext links to external databases such as CyanoBase, UniProt, and NCBI (Figure 1c). Furthermore, SynechoNET can be a reference site that allows the application of other cyanobacterial genomic resources to examine their protein-protein interaction network for a functional genomics study.

## Construction and content

### PSIMAP-based interactions

3,672 *Synechocystis *proteins were retrieved from UniProt (version 12) and were aligned with SCOP (version 1.69) domains using the PSI-BLAST algorithm with a common expectation value (E-value) cutoff of 0.001. By applying SCOP domain interaction pairs obtained from the PSIMAP-based interaction information database, PSIbase (build 3) [27], 12,748 predicted protein-protein interactions were obtained which involve 1,028 cyanobacterial proteins. They comprise 28% of all proteins in *Synechocystis*.

### Interactions based on iPfam, InterDom, and STRING

Pfam domains of all the *Synechocystis *proteins were collected from SwissPfam [17]. All proteins in *Synechocystis *were mapped to Pfam domain-interacting partners from iPfam (version 19), resulting in the construction of 13,448 predicted protein-protein interactions involving 1,541 proteins. They account for 42% of all proteins in *Synechocystis*. Likewise, *Synechocystis *proteins were mapped to Pfam domain-interacting partners from InterDom, resulting in 80,319 predicted protein-protein interactions involving 1,760 proteins. They account for 48% of all *Synechocystis *proteins. Furthermore, 2,658 proteins comprising 72% of all *Synechocystis *proteins were involved in 26,805 protein-protein interactions directly obtained from STRING (version 6.3). Taken together, SynechoNET revealed that 2,930 proteins participate in 109,532 protein-protein interactions. It is noteworthy that they comprise 79% of all proteins in *Synechocystis*.

### High-confidence interactions

To denote the confidence of the *in silico *prediction of protein-protein interactions, we used the number of databases that provide supporting evidence for each interaction as well as the reported reliability scores from InterDom and STRING. As a filter, we selected 509 *Synechocystis *proteins participating in 1,591 high-confidence protein-protein interactions that were commonly found in all the databases encompassing PSIMAP, iPfam, InterDom, and STRING. Those were further rescaled into the confidence range from 0.0 to 1.0 using the arithmetic means of InterDom and STRING scores. The resultant high-confidence protein-protein interaction network was dynamically visualized in Java applet viewer, a modified version of the public Integrator program [28].

### Transmembrane topology and domain structure

In addition to the interaction information of SynechoNET, it was reinforced to contain the information on membrane proteins that includes transmembrane topology, signal peptide, and domain structure information provided by Phobius and the prokaryotic version of Localizome program. The Localizome program gives an advantage for users to see the transmembrane topology and domain structure of cyanobacterial proteins at a glance.

## Utility and discussion

### Web interface

SynechoNET provides user-friendly web interfaces by (i) keyword search (Figure 1d) including gene name, gene locus name, GenBank ID, and UniProt entry name, (ii) functional category search (Figure 3a), and (iii) dynamic navigation of high-confidence protein-protein interactions (Figure 3d). A search result displays the list of high-confidence interaction partners of a query protein as well as the list of all the candidate interacting proteins. For each predicted interaction, it also accompanies supporting evidence, protein description, transmembrane and domain information, links to external databases, and their synonymous IDs (Figure 3b and 3c). On the same page, the list of high-confidence interaction partners is directly linked to an interactive network display highlighting those proteins. In addition, the buttons indicating the existence of supporting evidence are linked to popup windows displaying more detailed information such as interacting domains, domain positions, and direct link to the original web site. The information about transmembrane topology, signal peptide, and domain structure available from Phobius and Localizome is visualized by clicking the 'M' button in violet color indicating a membrane protein (Figure 3b, 3e, and 3f).

### Further experimental study and validation of SynechoNET

To validate SynechoNET, we examined the interactibility between histidine kinase and response regulators involved in *Synechocystis *positive phototaxis using yeast two-hybrid analysis. The result showed that the hybrid sensory kinase Sll0043 strongly interacts with cognate response regulators, Sll0038 and Sll0039 (data not shown). These experimental protein-protein interactions were consistent with the high-confidence prediction result of SynechoNET. On the other hand, in the analysis of membrane protein complexes of *Synechocystis*, we found evidence that photosystem II D2 protein (Sll0849) and cytochrome b6 protein (Slr0342) interact directly with photosystem D1 protein (Sll1867) and cytochrome b6f complex subunit 4 (Slr0343), respectively. Furthermore, the experimentally-verified nine transmembrane helices of MntB protein encoded by *sll1600 *[29] was also confirmed by the Phobius result provided in SynechoNET even though one of the nine transmembrane helices showed a weak signal in the probability profile of Phobius. Based on these experimental and bibliographic evidences, we suggest that the *in silico *protein-protein interaction and transmembrane topology information provided by SynechoNET is useful and reliable for the functional genomics study of *Synechocystis*.

## Conclusion

SynechoNET is a database and website that provides predicted protein-protein interactions. It integrates public protein-protein interaction databases that contain mutually complementary as well as redundant data. It is designed for biologists who are interested in the unicellular cyanobacterium *Synechocystis*. SynechoNET can be used for the analysis of regulatory membrane proteins by predicting transmembrane topology and domain structure. In particular, approximately one third of the *Synechocystis *proteome are left to be fully annotated. Thus, SynechoNET can help biologists to annotate them by analyzing their predicted interaction partners, membrane association, and membrane topology.

## Availability and requirements

SynechoNET is freely available at  and directly at . All the generated protein-protein interaction lists in tab-delimited and Cytoscape [30] formats can be found at . The dynamic interaction viewer based on Java applet technology requires Java-enabled web browsers.

## Competing interests

The authors declare that they have no competing interests.

## Authors' contributions

WK constructed the database, developed the website, and helped to draft the manuscript. SK designed the system, coordinated the project, and wrote the manuscript. BK helped to extract the interaction list. JO manually validated the interaction list. SC modified and improved the Java applet viewer. JB conceived and directed the study and helped to draft the manuscript. JC directed the project, participated in the system design, and helped to draft the manuscript. All authors read and approved the final manuscript.
